# Virtual Groups Address Visit-Associated Logistical Challenges but Require Dedicated Technical Support

**DOI:** 10.1093/geroni/igaf122.913

**Published:** 2025-12-31

**Authors:** Maureen O’Connor, Jaye McLaren, Andrew Nguyen, Kendra Pugh, Lauren Moo, Steven Shirk, Samantha Harrington

**Affiliations:** Bedford Veterans Health Care System, Bedford, Massachusetts, United States; VA Bedford Healthcare System, Bedford, Massachusetts, United States; Department of Veterans Affairs, Bedford, Massachusetts, United States; VA Bedford Health Care System, Bedford, Massachusetts, United States; VA Bedford Healthcare System, Bedford, Massachusetts, United States; VA Bedford Health Care System, Bedford, Massachusetts, United States; New England Geriatric Research, Education & Clinical Center, Bedford, Massachusetts, United States

## Abstract

The demanding nature of caregiver often acts as a barrier to accessing in-person care services and programs. Many caregivers find it difficult to travel to and from in-person support and education programs. Additionally, many caregivers are uncomfortable leaving the care recipient home alone and/or are unable to find respite care. These difficulties are often intensified for rural carers. For example, compared to their urban counterparts, rural dementia caregivers typically know of fewer available services and specialty providers and must travel significantly farther to find services. Telehealth services, provided via video or phone, can reduce these barriers. The current study adapted an in-person educational intervention to teach dementia caregivers how to manage neuropsychiatric symptoms to telehealth, named TeleCARE. The intervention took place over 8 weeks, 1 hour a week, over Webex. Twenty-four mostly white (100%), female (76%), spousal (82%) caregivers with an average age of 72 (M = 71.8, SD = 12.6) completed the study. Seventy percent of caregivers had some type of difficulty using telehealth technology and we quickly realized personalized help was necessary to support intervention engagement. Over the 8 sessions, less support was needed with each passing session as caregivers became more fluent with using the technology. At week 8, none of the caregivers required technology assistance. By the end of the intervention, caregivers reported a sense of accomplishment and increased comfort with the technology. At 3-month follow-up, technology support was again required. This talk will focus on the lessons learned as they are broadly applicable to telehealth interventions for dementia caregivers.

